# The dynamic connectome: towards large-scale 3D reconstruction of brain activity in real-time

**DOI:** 10.1186/1471-2202-14-S1-P407

**Published:** 2013-07-08

**Authors:** Xerxes D Arsiwalla, Alberto Betella, Enrique Martinez, Pedro Omedas, Riccardo Zucca, Paul FMJ Verschure

**Affiliations:** 1 SPECS, DTIC, Universitat Pompeu Fabra, Barcelona, Spain; 2 ICREA, Barcelona, Spain

## Introduction and methods

**Figure 1:**
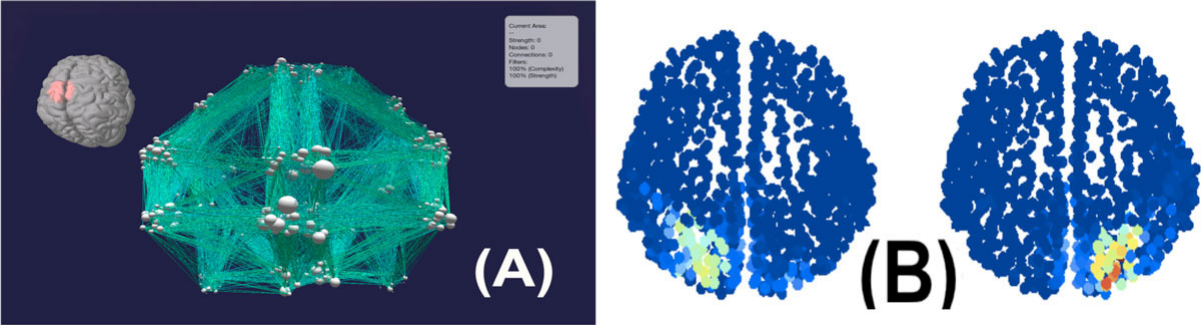
**(A) snapshot of the cortical connectome network in virtual reality**. (B) activation in the parietal lobes just after stimulation of the superior parietal area. Activity persists for about 5 secs after stimulation.

What does large-scale connectivity tell us about whole-brain activity and neural circuits? In this work, we present a virtual reality based large-scale dynamic simulation for 3D reconstruction of whole-brain activity over the cortical connectome in real-time. Using DTI structural connectivity data from [1] we built an interactive 3D visualization of the human connectome network in an immersive virtual reality environment (Figure 1A) using the Unity 3D gaming engine. Further, the virtual reality brain network in Unity is coupled to a real-time neuronal simulator, iqr [2]. As we see, coupling structural connectivity data with detailed enough neuronal population dynamics is sufficient in predicting functional correlations and large-scale activity patterns. We model neuronal dynamics by a linear-threshold filter (as work in progress, we are currently implementing population dynamics from mean-field models [3]). Each population module is stochastic, having Gaussian noise. The user can stimulate any region or simultaneous regions of the network with external input currents. The simulation then reconstructs reverberating neural activity propagating throughout the network in real-time. As an explicit example, we stimulate the superior parietal areas and observe causal activity propagation in the parietal lobe, indicative of visuo-motor integration (Figure 1B). This is a first step to simulating and mapping large-scale brain activity in real-time.

## Results and conclusions

As quantitative analysis methods and data-recording technology in neuroscience make improvements, it is becoming evident large-scale dynamics and whole-brain quantitative measures play an important role. For instance, oscillations across large brain regions are precursors to several cognitive functions. Moreover, the causal map in these interactions is crucial. Compared to functional correlations, large-scale temporal activity maps across directionally connected brain structures serve as a more powerful tool to unravel mechanisms of large-scale neural circuits. Our results show that stimulating brain areas triggers a sequence of causal activations in associated network loops that represent cognitively related functions.
